# *FtbZIP85* Is Involved in the Accumulation of Proanthocyanidin by Regulating the Transcription of *FtDFR* in Tartary Buckwheat

**DOI:** 10.3390/cimb45040221

**Published:** 2023-04-13

**Authors:** Shuangshuang Liu, Jianmei Wang, Zhibin Liu, Yi Yang, Xiaoyi Li

**Affiliations:** Key Laboratory of Bio-Resources and Eco-Environment of Ministry of Education, College of Life Sciences, Sichuan University, Chengdu 610065, China

**Keywords:** ABA, *FtbZIP85*, *FtSnRK2.6*, hairy roots, proanthocyanidin, Tartary buckwheat

## Abstract

As a drought-tolerant crop, Tartary buckwheat survives under adverse environmental conditions, including drought stress. Proanthocyanidins (PAs) and anthocyanins are flavonoid compounds, and they participate in the regulation of resistance to both biotic and abiotic stresses by triggering genes’ biosynthesis of flavonoids. In this study, a basic leucine zipper, basic leucine zipper 85 (*FtbZIP85*), which was predominantly expressed in seeds, was isolated from Tartary buckwheat. Our study shows that the expressions of *FtDFR*, *FtbZIP85* and *FtSnRK2.6* were tissue-specific and located in both the nucleus and the cytosol. FtbZIP85 could positively regulate PA biosynthesis by binding to the ABA-responsive element (ABRE) in the promoter of dihydroflavonol 4-reductase (*FtDFR*), which is a key enzyme in the phenylpropanoid biosynthetic pathway. Additionally, FtbZIP85 was also involved in the regulation of PA biosynthesis via interactions with FtSnRK2.6 but not with FtSnRK2.2/2.3. This study reveals that *FtbZIP85* is a positive regulator of PA biosynthesis in TB.

## 1. Introduction

Tartary buckwheat (*Fagopyrum tataricum*, TB) is a common plant in the family Polygonaceae, which originates from southwest China [1]. TB contains not only abundant nutrients but also high contents of bioactive flavonoids, including proanthocyanidins (PAs), anthocyanins, rutin and quercetin [2], and these flavonoids have been largely used as over the counter nutritional supplements [3]. PAs, as flavonoid compounds, participate in the regulation of biotic and abiotic stresses. Previous studies have shown that transcription factors regulate PA synthesis by regulating the expressions of related genes in the flavonoid synthesis pathway [4,5,6]. For example, in *Fagopyrum tataricum,* a v-myb avian myeloblastosis viral oncogene homolog 8 (*FtMYB8*)*, FtMYB18* and *FtMYB3* act as negative regulators in PA/anthocyanin biosynthesis [7]. Abscisic acid (ABA) signaling is involved in PA biosynthesis in the persimmon fruit of *Diospyros kaki* via *DkMYB4* activation by DkbZIP5 [8]. MdbHLH33, an apple bHLH transcription factor (TF) in *Malus* × *domestica*, by activating MdCBF2 and MdDFR expression, was identified as affecting cold tolerance and anthocyanin accumulation of the positive adjustment factor [9]. Meanwhile, dihydroflavonol 4-reducase (DFR) acts as a key hub in the flavonoid synthesis pathway, directly affecting the biosynthesis of PA [10]. DFRs are a family of NADPH-dependent reductases [11,12]. Many species have been found with *DFR* with different kinds of regulatory regions, which can interact with many TFs or proteins to regulate *DFR’* expressions [13]. Moreover, exogenous ABA can affect the expression level of *DFR* [14].

As sessile organisms, plants have a sophisticated regulatory mechanism to regulate their internal environmental homeostasis to adapt to external environmental changes and cope with various biological stresses. ABA plays an indispensable role in plant stress resistance [15,16]. SnRK2.6 has been identified as a serine (S)/threonine (T) protein kinase in Arabidopsis, and it is activated via the phosphorylation of amino acid (aa) residue(s) within the activation loop [17,18]. SnRK2s can phosphorylate AREB/ABFs (ABA-responsive element-binding proteins), a group of basic leucine zipper (bZIP) transcription factors, which activate the expressions of downstream ABA-responsive genes [19]. ABA response kinase substrate 1 (AKS1), a basic helix–loop–helix (bHLH) transcription factor in Arabidopsis, is phosphorylated by SnRK2.6 [20]. The bZIP TFs of the ABA-responsive element-binding factor family (ABF), also known as ABRE, regulate the transcription of ABA-induced genes, and they have been considered the true substrates of SnRK2s [21]. bZIP proteins regulate the expressions of downstream genes by binding to ACGT core cis-elements, such as ABRE, G-box (CACGTG), C-box (GACGTC), A-box (TACGTA), AAGCT (T-box), the GCN4 motif and TGA(G/C) TCA [22]. In addition, bZIP TFs have also been shown to be involved in PA biosynthesis metabolic pathways [23,24,25]. For example, *MdBB*×*20* promotes anthocyanin biosynthesis when overexpressed in *apple calli* [10]. MdHB1, the HD-Zip I TF, indirectly inhibits the transcription of *MdDFR* and *MdUFGT* (UDP-flavonoid glucosyltransferase) by inhibiting the entry of MdMYB10, MdbHLH3 and MdTTG1 into the cytoplasm. The silencing of *MdHB1* results in the activation of *MdDFR* and *MdUFGT*’ expression by these transcription factors and promotes anthocyanin biosynthesis [24]. Additionally, *MdbZIP44* regulates ABA-promoted anthocyanin accumulation, which causes apple pulp discoloration [22].

In this study, we identified a bZIP transcription factor, *FtbZIP85*, in TB. We found that FtbZIP85 was located in the nucleus and exclusively expressed in seeds. FtbZIP85 could bind the BZIP(CACGTG) element of the promoter region of *FtDFR*. Dual-Luc assays showed that FtbZIP85 could activate *FtDFR’* expression. In addition, FtbZIP85 could interact with FtSnRK2.6 but not with FtSnRK2.2/2.3 in vitro and in vivo.

## 2. Materials and Methods

### 2.1. Plant Materials and Growth Conditions

In this study, the TB variety “ChuanQiao NO.3” was used, cultivated by the Xichang Academy of Agricultural Sciences. Experimental materials were planted in a greenhouse for plant tissue culture at 22 °C, with light time set to: 16 h light/8 h dark cycle. The soil used in the experiment was mixed with vermiculite and nutrient soil at a ratio of 1:3.5 and sterilized. Murashige and Skoog (MS) medium (Murashige and Skoog, 1962) supplemented with 3% (*w*/*v*) sucrose and 1.2% (*w*/*v*) agar, with pH 5.8, was used for sterile tissue culture.

### 2.2. Chemicals

Most of the chemicals were purchased from Sigma Aldrich (http://www.sigmaaldrich.com/, accessed on 20 October 2020); the others were obtained from Sangon (Shanghai, China).

### 2.3. Hairy Root Induction

The primary steps were carried out according to a previously published protocol [26]. The steps to note are as follows: (1) Tartary buckwheat seeds were soaked in water for approximately 1 h (Note: At this time, the Tartary buckwheat seeds skin are the easiest to peel); (2) Tweezers were used to peel off the black brown skin of seeds and put them into a 10 mL centrifuge tube, approximately 1/5 of which should be reserved; (3) After germination, when the first true leaf has not yet grown or the cotyledon has just been extended completely, the cotyledon and its hypocotyl parts were cut as explants for hairy root culture; (4) Selected explants with good growth segments were completely immersed in *A. rhizogenes* solution for 10 to 15 min, and gently shaken to make the floating explants fully impregnated; (5) The well-grown hair roots obtained by resistance screening were cut off with a sterilized scalpel, transferred to the hair root culture plate for further culture and marked. After a certain amount of root growth, a part of the root was cut for PCR identification; (6) During the period, the medium was changed every week, the sterile operation was noted and the contaminated hair roots were cleaned in time.

### 2.4. ABA Treatment Assay

After approximately 2 months, the hairy roots grew well. In order to compare ABA tolerance in normal and transgenic lines, these hairy roots were cultured in MS containing various concentrations of ABA (50 μM, 75 μM). The experiment was performed in a completely randomized manner with three replications, and samples were taken at 0 d, 1 d, 3 d, 6 d and 9 d after treatment for analyses.

### 2.5. RNA Extraction for RT-qPCR Analysis

For a gene expression analysis, the tissues, including the roots, stems, cotyledon, leaves and immature seeds, were harvested from TB for RNA extraction according to the method described in [27]. First-strand cDNA was synthesized using a PrimeScript^TM^ RT Master Mix (Perfect Real Time) (Takara, Otsu, Shiga, Japan) according to the manufacturer’s instructions. A Bio-Rad CFX96 RT-PCR type of machine was used for RT-qPCR assay, and SYBR Premix Ex Taq II (Takara, Otsu, Shiga, Japan) was the reagent. The relative mRNA levels were calculated using the comparative CT method, which was normalized against the ACTIN [23] expression in the same sample. The primers are listed in Table S1.

### 2.6. Measurement of Metabolites Using High-Performance Liquid Chromatography

Flavonoids were extracted according to the method described previously [28], with some modifications. The samples were freeze-dried in vacuum and ground with ceramic beads at 50 Hz for 5 min with a mixing mill (MM 400, Retsch) to a fine powder. Sample powder (0.1 mg) was extracted with 1.0 mL 80% methanol in a −20 °C refrigerator for 12 to 16 h. This was then centrifuged at the maximum speed of the centrifuge for 10 min at 4 °C and repeat if impurities remain. The supernatant was filtered with a 1 mL sterile syringe combined with a 0.22 µm microporous organic filter membrane (SCAA-104, 0.22 mm pore size; ANPEL, Shanghai, China, http://www.anpel.com.cn/, accessed on 17 September 2021) and stored in a brown bottle at −80 °C for LC-MS/MS analysis (the concentration and loading amount of sample were adjusted according to the content of compounds). Standards and reagents were all before the experiment, and the samples were sorted and submitted to the college platform for compound determination and analysis. Analysis was conducted on three independent biological replicates, each containing three technical replicates. 

### 2.7. Phylogenetic Analysis 

A phylogenetic analysis of *FtDFR* was carried out, and another reductase, *SnRK2.6*, from different plant species was analyzed using the same method. The sequences were downloaded from the UniProt database (https://www.uniprot.org, accessed on 26 April 2022) and the National Center for Biotechnology Information nonredundant protein database based on protein–protein alignment tools (https://blast.ncbi.nlm.nih.gov/Blast.cgi, accessed on 26 April 2022) with their proteins as the bait. Evolutionary analyses were conducted in MEGA X [29], and the Evolview (Evolview: Home (evolgenius.info)) website was used to beautify the evolutionary tree. The maximum likelihood method and the model based on JTT matrix [30] were used to construct the evolutionary tree.

### 2.8. Conserved Motifs of the Key Genes in TB Flavonoid Synthesis Pathway

Conserved motifs were also analyzed for the key genes’ proteins in the TB flavonoid synthesis pathway using MEME software. In total, 20 motifs were identified, as shown in Supplemental Figure S1. The ID of the key genes analyzed in the TB flavonoid synthesis pathway are listed in Table S2.

### 2.9. Subcellular Localization 

To determine the subcellular localizations of FtbZIP85, FtDFR and FtSnRK2.6, the CDS of *FtbZIP85* was cloned into the pBI121-eGFP plasmid. The CDSs of *FtDFR* and *FtSnRK2.6* were cloned into the pBI121-mCherry plasmid separately. The recombinant plasmids were transformed into *N. benthamiana* leaves via Agrobacterium-mediated transformation. All the leaves were collected 36 h after transformation, and the fluorescent signals were detected using a confocal laser-scanning microscope (DMI6000B; Leica, Mannheim, Germany).

### 2.10. EMSA

The CDS sequence of *FtbZIP85* was inserted into the protein expression vector pGEX6p-1 and transformed into the *Escherichia coli Rosetta* (DE3) cells (Novagen, Darmstadt, Germany), which were oscillated and cultured overnight at 37 °C. The prokaryotic expression and purification methods of the protein were described in the instructions for the molecular cloning experiment. The GST-FtbZIP85 (glutathione S-transferase) protein was optimally induced at 16 °C, with an IPTG concentration of 0.6 mM, and the molecular weight of the fusion protein is approximately 34 KDa. Four ABRE motifs were found in the prediction of the *FtDFR* regulatory regions. Therefore, double-stranded DNA containing four ABRE elements and 15 bases at both ends of the upstream and downstream of *FtDFR* and double-stranded DNA with mutated motifs were designed and synthesized. The oligonucleotide probes were synthesized. In terms of probe-mBZIP, ABRE was mutated as the negative control. An EMSA kit (Thermo Scientific, China, catalog number: 20148) was used, and detection was carried out using Image Lab 2.0 (Bio-Rad, Hercules, CA, USA). The probes for EMSA are listed in Table S1.

### 2.11. Y1H Assay 

According to the user manual, Clontech’s Matchmaker Gold Yeast One-Hybrid Library Screening System was used to verify whether the FtZIP85 protein could bind to the ABRE motif on *FtDFR* regulatory regions. The pAbAi vector was used to construct the bait vector, the pGADT7 vector was used to construct the prey vector and the ABRE motif in the *FtDFR* regulatory regions was inserted into the minimum promoter region upstream of reporter AUR1-C (gold BA resistance gene) as bait. The CDS sequence of the *FtZIP85* protein was inserted into the pGADT7 vector as prey. If there was interaction between them, the yeast (Y1H Gold) transformed strain could grow on the SD medium without uracil Ura, leucine Leu and gold basinin aureobasidin A (AbA), otherwise it could not grow. 

### 2.12. Dual-Luc Assay

A transient double luciferase reporting (LUC) assay was used to further confirm whether FtbZIP85 promoted the activation or inhibited the transcription of the *FtDFR* gene. The regulatory region of the *FtDFR* gene at −2 k bp upstream of ATG was amplified and cloned into the pGreenⅡ0800-LUC (Reporter) vector. The CDS sequence of FtbZIP85 (excluding the stop codon) was amplified and cloned into the pCambia1300-eGFP (Effector) vector. After the successful construction, all vectors were transformed into the *Agrobacterium* strain GV3101 in the presence of the helper plasmid pSoup-p19. The dye solution formula was: 10 mM MgCl_2_/10 mM MES. Agrobacterium-suspensions were mixed separately at a ratio of 1:1 and injected into *tobacco* leaves. Samples were taken after 2.5 d of continued culture and used for LUC experiments. The dual luciferase detection system (Promega, Madison, WI, USA) was used according to the instructions provided by the evaluation of the ratio between LUC and REN.

### 2.13. Y2H Assay

To verify the interactions between FtbZIP85 and FtSnRK2s, the CDSs of *FtSnRK2s* were amplified and inserted into pGADT7 as baits. The coding region of *FtbZIP85* was cloned into pGBKT7 (Clontech, Mountain View, CA, USA) as the prey. The bait and prey constructs were co-transformed into the yeast strain AH109, using the previously described method [31]. The yeast strains were grown on a selection medium lacking SD/-Trp/-Leu and SD/-Trp/-Leu/-His/-Ade. In addition, an FtbZIP85-pGBKT7 vector was constructed, transferred into Y2H and cultured on an SD/-Leu/-Trp and SD/-Leu/-Trp/X-Gal strain medium at 30 °C to verify the autonomous activation activity. The transcriptional autoactivation of FtbZIP85-pGBKT7 was determined by the bluing of *β*-galactose in X-α-gal stain yeast cells.

### 2.14. BiFC Assay

The interaction between FtbZIP85 and FtSnRK2.6 was confirmed by the bimolecular fluorescence complementation (BiFC) assay. The CDS of *FtSnRK2.6* were cloned into the vectors containing the C-terminus of the yellow fluorescent protein (YFP). The CDS of *FtbZIP85* was cloned into the vector containing the N-terminus of YFP. Then, various nYFP-FtbZIP85 and cYFP-FtSnRK2.6 fusion vectors were introduced into the *A. tumefaciens* strain GV3101. After incubation, the Agrobacterium cells were harvested and resuspended in an infiltration buffer (0.2 mM acetosyringone, 10 mM MgCI_2_ and 10 mM MES) at identical concentrations (OD_600_ = 0.5), and then equal amounts of the combined plasmids were transformed into *N. benthamiana* leaves via Agrobacterium-mediated transformation. All plants were incubated in a growth chamber at 24 °C. The YFP signals were collected and detected with a confocal laser-scanning microscope (DMI6000B; Leica, Mannheim, Germany).

### 2.15. Pull-Down Assay

To test the interaction between FtbZIP85 and FtSnRK2.6, the CDS of *FtbZIP85* was cloned into the pGEX-6p-1 plasmid (Amersham, Piscataway, NJ, USA) to produce FtbZIP85-GST, and the CDS of *FtSnRK2.6* was inserted into the pMal-p2x plasmid to produce FtSnRK2.6-MBP. The recombinant proteins were induced using 0.5 mM isopropyl-β-dthiogalactopyranoside (IPTG) for 16 h at 16 ℃ into *Escherichia coli* Rosetta (DE3) cells (Novagen, Darmstadt, Germany). The proteins were purified using amylose magnetic beads (Sangon Biotech) or glutathione resins (GS4B; GE Healthcare, Pittsburgh, PA, USA). 

For a GST pull-down assay, FtSnRK2.6-MBP and FtbZIP85-GST recombinant proteins were incubated in 50 μL GST beads in a GST-binding buffer (50 mM Tris-HCI, pH 7.5, 100 mM NaCI, 1 mM ethylenediaminetetraacetic acid (EDTA), 0.05% 3-mercaptoethanol and 0.2% Triton X-100) for 2 h at 4 °C. Subsequently, the mixture was washed three times with a GST wash buffer (550 mM Tris-HCI, pH 7.5, 100 mM NaCl, 1 mM EDTA, 0.05% p-mercaptoethanol and 1% Triton X-100) for 10 min. The bound proteins were boiled for 10 min in a 1 × sodium dodecyl sulfate–polyacrylamide gel electrophoresis (SDS-PAGE) (1 × SDS) loading buffer, separated using 10% SDS-PAGE and then visualized using immunoblot assays with anti-GST and anti-MBP antibodies (Santa Cruz, TX, USA). Western blots were performed using the ECL chemiluminescence detection system (Bio-Rad, Hercules, CA, USA).

### 2.16. Co-IP Assay

Co-immunoprecipitation (Co-IP) assay was performed as described [31]. The CDS of FtbZIP85 was cloned into pBI121-eGFP and the CDS of FtSnRK2.6 was cloned into the vector of pCAMBIA-2300-5Flag, respectively. Different combinations of Agrobacterium were transformed into *tobacco* cells for expression. The protein lysis and extraction buffer was formulated as follows: 50 mM HEPES, pH 7.5, 150 mM NaCI, 1 mM EDTA, 0.1%SDS, 1%Triton X-100 (*v*/*v*), 0.1% deoxycholate (*w*/*v*) and EDTA-free protease inhibitor cocktail (#4693132001, Roche). The protein sample of the cracking and extraction buffer was incubated for 30 min on the ice. After the liquid was mixed, the mixtures were separated in the centrifuge tube at the maximum speed for 30 min at a low temperature. The supernatant was then filtered with a layer of nylon membrane gauze (to further remove impurities). After cryogenic centrifugation, anti-Flag, in the extraction of the suspension^®^ M2 magnetic beads (Sigma-Aldrich), was put into rotating incubation at 4 °C for 6 to 7 h. The supernatant was discarded by centrifugation and rinsed three times with phosphate-buffered saline (PBS, pH 7.5) for 10 min each time. Following cryogenic centrifugation, 5 × SDS loading buffer were added to the denatured proteins. SDS-PAGE was used to separate the proteins. Immunoprecipitation was detected by the anti-Flag antibody and anti-GFP antibody, respectively.

### 2.17. In Vitro Phosphorylation Assay

The proteins of FtbZIP85-GST and FtSnRK2.6-MBP were purified as previously described. For an in vitro kinase assay, the protein samples were mixed in a reaction buffer (50 mM Tris-HCI at pH 7.5, 10 mM MnCI_2_, 10 mM MgCI_2_, 1 mM DTT and 10 mM ATP), which was conducted as previously described. The kinase reaction was conducted at 30 °C for 1.5 h. The reaction mixtures were terminated with 2 × SDS loading buffer. The subsequent experimental methods refer to the above Co-IP assay, and the antibody was replaced with protein phosphothreonine antibody (Cell Signaling Technology, Beverly, MA, USA) for the detection of the target.

### 2.18. Statistical Analysis

All data were analyzed by performing an analysis of variance using GraphPad Prism 9 (GraphPad Prism 9, Incorporated, Boston, MA, USA) and SPSS (SPSS28, Incorporated, San Jose, CA, USA). According to the results of the ANOVA analysis with the Tukey test, different letters indicate significant differences (*p* < 0.05). Adobe Photoshop 2020 (CS20, Adobe Systems Incorporated, San Jose, CA, USA) and Adobe Illustrator (CS6, Adobe Systems Incorporated, San Jose, CA, USA) were used to prepare the figures.

## 3. Results

### 3.1. Expression Profile Analysis of FtbZIP85 and PA Biosynthesis-Related Genes

**Figure 1:**
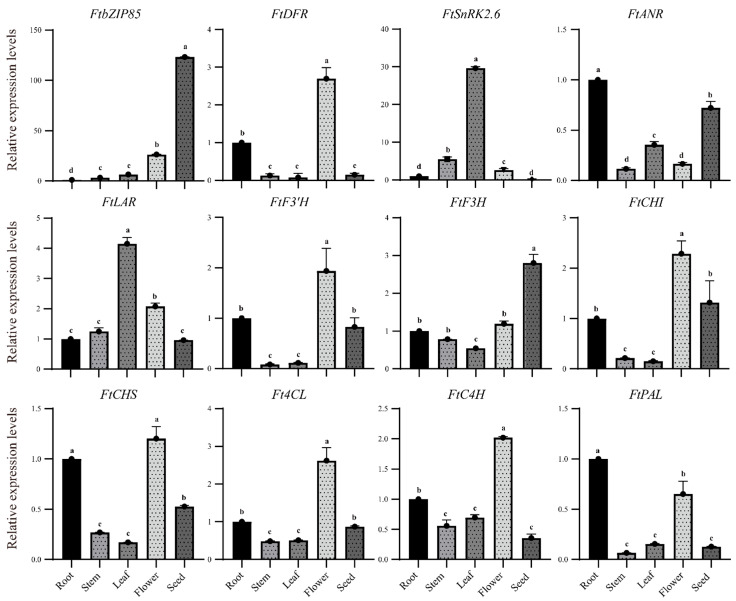
The expression patterns of FtbZIP85 and PA biosynthesis-related genes in different tissues. The RNA transcript levels of FtbZIP85 and the PA biosynthesis-related genes in different tissues were analyzed using an RT-qPCR assay. Values are means ± SD (n = 3). The gene expression level in the roots was defined as “1”. Bars represent the standard deviations of three independent experiments. According to the results of the ANOVA analysis with the Tukey test, different letters indicate significant differences (*p* < 0.05). The enzyme names or genes and flavonoid compounds are abbreviated as follows: DFR, dihydroflavonol 4-reductase; ANR, anthocyanidin reductase; LAR, leucoanthocyanidin reductase; F3′H, flavonoid 3′-hydroxylase; F3H, flavanone 3-hydroxylase; CHI, chalcone isomerase; CHS, chalcone synthase; 4CL, 4-coumarate: CoA ligase; C4H, cinnamic acid 4-hydroxylase; PAL, phenylalanine ammonia lyase.

Our previous study showed that FtbZIPs are similar to Arabidopsis bZIP ABI5, which is an important regulator in ABA signaling [25]. In addition, FtZIP5 interacts with FtSnRK2.6 [32]. In this study, we found that *FtZIP85* was notably expressed in the seeds (Figure 1). Additionally, we also investigated the expression pattern of *FtSnRK2.6* and the key genes in PA biosynthesis using RT-qPCR (Figure 1). *FtSnRK2.6* showed the highest expression level in the leaves, and it showed the lowest expression level in the seeds. The transcript levels of *FtDFR*, *FtF3′H*, *Ft4CL*, *FtC4H* and *FtCHI* in the flowers were higher than those in the other tissues. In addition, the transcript levels of *FtANR* and *FtPAL* were the lowest in the roots, and the transcript levels of most of the PA biosynthesis-related genes (*FtDFR*, *FtF3′H*, *FtCHI*, *FtCHS*, *Ft4CL* and *FtC4H*) were lower in the stems and the leaves but higher in the flowers. 

### 3.2. FtbZIP85 Activates the Expression of FtDFR by Binding to Its Promoter

**Figure 2:**
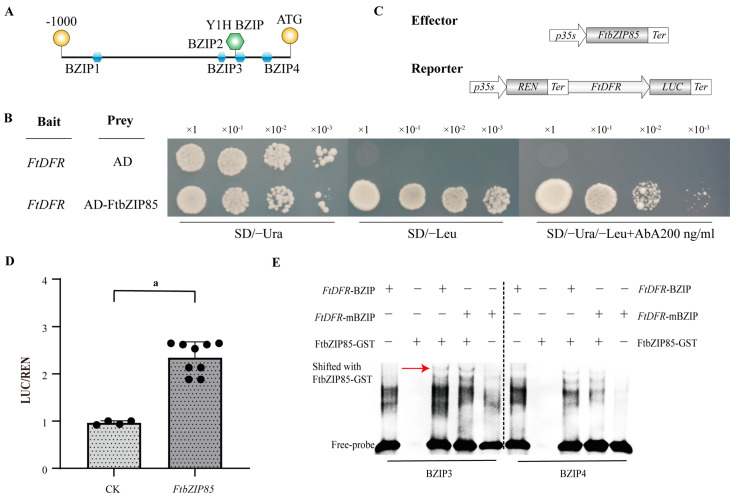
FtbZIP85 promoted the transcriptional activation of FtDFR. (**A**) Location of the motifs of BZIP, BZIP1, BZIP2, BZIP3 and BZIP4 in the *FtDFR* promoter, including ABRE cores (ACGTA/T/C) and mBZIP, mutant probe of BZIP. (**B**) Y1H assay showing that FtbZIP85 binds ABRE in the *FtDFR* promoter. The sequences containing intact BZIP (Y1H BZIP) were cloned into the pAbAi plasmid. The transformed yeast clones were grown to the cell concentration of OD_600_ = 1.0. Serial dilutions (1:1, 1:10, 1:100, 1:1000) of the transformed yeast cells were incubated on a plate lacking SD/-Leu with or without AbA. The AbA concentration was 200 ng/mL. (**C**) Schematic diagram showing the LUC vector. (**D**) Transactivation of the *FtDFR* promoter by FtbZIP85. The *FtDFR* promoter sequence was used to drive the firefly LUC gene, creating the reporter *FtDFR* pro-LUC. The relative ratio of LUC/REN was determined in TB leaves by co-transforming the reporter plasmids with the effector construct. CK is an empty vector. Error bars represent ± SD of three biological replicates. According to the results of the ANOVA analysis with the Tukey test, different letters indicate significant differences (*p* < 0.05). (**E**) EMSA showing the specific binding of FtbZIP85 to BZIP in the *FtDFR* promoter. The normal and mutant probes (m) were labeled with biotin. Unlabeled intact probes were used for competition. FtDFR-BZIP are competing probes. FtDFR-mBZIP are mutated competing probes. The red arrows indicate the shifted band representing the protein–DNA complex. The plus (+) and minus (−) signs denote the presence and absence, respectively, of the probe or protein.

Next, we analyzed the 2k bp promoter sequence upstream of the key genes in the TB flavonoid synthesis pathway (Supplemental Figure S1). After a comprehensive analysis, we found that the sequence between −300 and −350 bp upstream of the ATG on the *FtDFR* promoter (Figure 2A) contained four BZIP motifs (Figure 2A) [33]. We performed a yeast one-hybrid (Y1H) assay to test whether FtbZIP85 could recognize the motifs in the *FtDFR* promoter. The results show that FtbZIP85 could bind to the bZIP/ABRE motif (BZIP) in the *FtDFR* promoter (Figure 2B), including BZIP3 and BZIP4 elements. Then, 200 ng/mL of AbA was used to inhibit the growth of the FtDFR-pAbAi “bait” yeast strain (Supplemental Figure S2A). In addition, FtbZIP85 was verified to have no self-activating activity (Supplemental Figure S2B), and it was found that BZIP1/2 were not the binding sites of FtbZIP85 (Supplemental Figure S2C). To test the transactivation activity of the *FtDFR* promoter via FtbZIP85, a Dual-Luc assay was performed, and the results demonstrated that FtbZIP85 could trigger *FtDFR* promoter transactivation (Figure 2D). To confirm the finding that FtbZIP85 directly binds BZIP in the promoter of *FtDFR*, we performed an electrophoretic mobility shift assay (EMSA). Oligonucleotides containing the BZIP label were used to examine the binding affinity. A retarded band was visualized in BZIP3 and BZIP4 but not in BZIP1 or BZIP2 (Supplemental Figure S2C). However, the phylogenetic analysis revealed that the evolutionary tree of DFRs was divided into eight large branches, and FtDFR was clustered with the other four genes of TB in one branch, indicating that the evolution of *FtDFRs* was relatively conserved (Supplemental Figure S3A) [30].

### 3.3. Subcellular Localization Analysis of FtbZIP85, FtDFR and FtSnRK2.6

**Figure 3:**
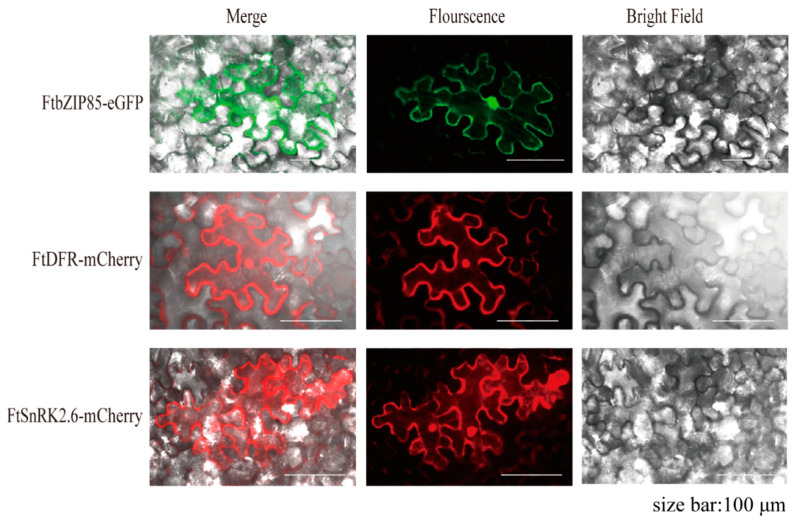
Subcellular localization analysis of FtbZIP85, FtDFR and FtSnRK2.6.

To explore the subcellular localizations of FtbZIP85, FtDFR and FtSnRK2.6 in *N. benthamiana* leaves, the full-length CDSs of *FtbZIP85*, *FtDFR* and *FtSnRK2.6* were fused with the vectors of pBI121-eGFP and pBI121-mCherry to obtain FtbZIP85-eGFP, FtDFR- mCherry and FtSnRK2.6-mCherry, respectively. The transient expressions of the fusion constructs in *N. benthamiana* leaves showed that eGFP/mCherry signals were detected in both the nucleus and the cytosol (Figure 3).

### 3.4. FtbZIP85 Is Phosphorylated by FtSnRK2.6

**Figure 4:**
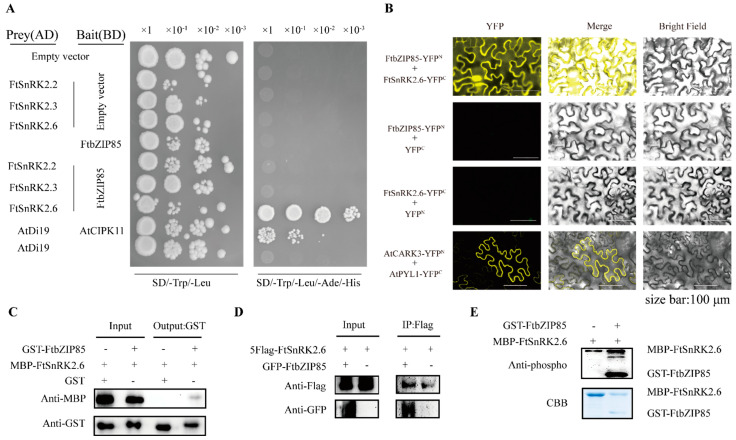
FtSnRK2.6 physically interacted with and phosphorylated FtbZIP85. (**A**) Y2H assay demonstrating the interaction between FtbZIP85 and FtSnRK2s. The full CDSs of *FtSnRK2s* were cloned into the pGADT7 plasmid, yielding FtSnRK2.2-AD, FtSnRK2.3-AD and FtSnRK2.6-AD. The CDS of *FtbZIP85* was cloned into pGBKT7, yielding FtbZIP85-BD. Serial dilutions (1:1, 1:10, 1:100, 1:1000) of transformed yeast cells were grown on SD/-Leu/-Trp and SD/-Leu/-Trp/-His/-Ade medium. (**B**) BiFC assay showing the interaction between FtbZIP85 and FtSnRK2.6. Bars = 100 μm. (**C**) Pull-down assay showing the interaction between FtbZIP85 and FtSnRK2.6. Purified FtbZIP85-GST or GST proteins were immunoprecipitated with GST beads. Immunoprecipitated proteins were incubated with FtSnRK2.6-MBP, and anti-MBP antibody was used to detect FtSnRK2.6-MBP. (**D**) Co-IP showing the interaction between FtbZIP85 and FtSnRK2.6. Total proteins were extracted from *N. benthamiana* leaves, where FtbZIP85-eGFP and FtSnRK2.6-5Flag fusion proteins were co-expressed. FtSnRK2.6-5Flag proteins were immunoprecipitated with Flag agarose beads and detected using anti-GFP and anti-Flag antibodies. (**E**) Phosphorylation of FtbZIP85 by FtSnRK2.6 in vitro. In vitro phosphorylation assay of FtbZIP85 was conducted via immunoblotting with an anti-phosphothreonine antibody.

According to the phylogenetic analysis of FtSnRK2s, a total of six large branches were clustered (Supplemental Figure S3B). FtSnRK2.6 and tr|A0A8K0YGH5| were clustered in a small branch, while FtSnRK2.2/2.3 were clustered in another large branch, indicating that *FtSnRK2.6* and *FtSnRK2.2/2.3* may play differential functions in TB. A previous report showed that FtZIP5 could interact with FtSnRK2.6. To assess the relationship between FtbZIP85 and FtSnRK2s, we performed Y2H experiments. The control experiments showed that the yeast strains with FtSnRK2s-AD or FtbZIP85-BD failed to grow on the SD/-Leu/-Trp/-His/-Ade medium. Afterward, FtbZIP85-BD was co-transformed into a yeast strain with FtSnRK2.6/2.2/2.3. The results show that *FtbZIP85* interacted with *FtSnRK2.6* but not with *FtSnRK2.2/2.3*. In addition, the FtSnRK2.6-MBP fusion proteins were pulled down by FtbZIP85-glutathione-S-transferase (GST) but not by GST alone, indicating that FtbZIP85 and FtSnRK2.6 had protein–protein interactions in vitro (Figure 4C). To test the interactions in vivo, a biomolecular florescence complementation (BiFC) assay was performed. FtSnRK2.6 and FtbZIP85 were fused with the C-terminus and N-terminus of the split yellow fluorescent protein (YFP), yielding the FtSnRK2.6-YFP^c^ and FtbZIP85-YFP^n^ constructs, respectively. We co-expressed the two constructs in *N. benthamiana* leaves and observed strong fluorescence signals in tobacco leaf epidermal cells, while no visible signals were produced in the control group (Figure 4B). Subsequently, we developed FtSnRK2.6-5Flag and FtbZIP85-eGFP fusion constructs and co-injected them into *N. benthamiana* leaves to conduct a co-immunoprecipitation (Co-IP) assay. The results show that an obvious FtbZIP85-eGFP band was detected in the anti-Flag immunoprecipitates from the leaves co-expressing the FtbZIP85-eGFP and FtSnRK2.6-5Flag fusion proteins (Figure 4D). Next, protein kinase experiments were performed. When FtbZIP85-GST and FtSnRK2.6-MBP co-existed, a 34 KDa FtbZIP85-GST phosphorylation band could be found, indicating that FtSnRK2.6 phosphorylated FtbZIP85 in vitro (Figure 4E). Overall, we demonstrated that FtbZIP85 interacted with and was phosphorylated by FtSnRK2.6.

### 3.5. ABA Is Involved in PA Biosynthesis by Regulating the Transactivation of FtDFR

**Figure 5:**
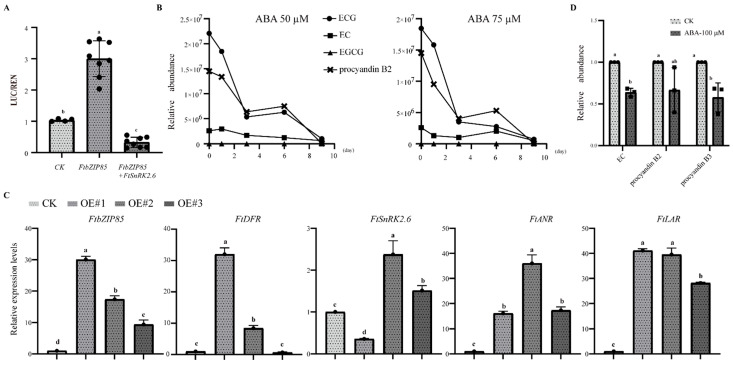
(**A**) Exogenous addition of *FtSnRK2.6* inhibited the transcriptional activation of FtbZIP85 to the *FtDFR’* promoter. The *FtDFR* promoter sequence was used to drive the firefly LUC gene, creating the reporter *FtDFR* pro-LUC. The relative ratio of LUC/REN was determined in TB leaves by co-transforming the reporter plasmids with the effector construct. CK is an empty vector. Error bars reflect ± SDs of three biological replicates. According to the results of the ANOVA analysis with the Tukey test, different letters indicate significant differences (*p* < 0.05). (**B**) Effects of ABA on PA biosynthesis in TB hairy roots. Samples were collected after sequential treatments for 0, 1, 3, 6 and 9 days. (**C**) Key gene expressions of PA biosynthetic pathway in TB hairy roots. CK is the control group; OE#1/#2/#3 are the different transgenic line expressions. The gene expression level in CK was defined as “1”. According to the results of the ANOVA analysis, different letters indicate significant differences (*p* < 0.05); error bars reflect ± SDs. (**D**) EC, procyandin B2 and procyandin B3 relative abundances in TB after ABA treatment. The abundance level in CK was defined as “1”. According to the results of the ANOVA analysis with the Tukey test, different letters indicate significant differences (*p* < 0.05); error bars reflect ± SDs.

In an attempt to analyze whether *FtSnRK2.6* has an effect on the transcriptional activation between *FtbZIP85* and *FtDFR*, we performed a luciferase complementation (LUC) assay. The co-expression of *FtSnRK2.6* and *FtbZIP85* with the reporter *FtDFR* pro-LUC resulted in a significantly decreased signal compared with that of the control (Figure 5A), indicating that FtSnRK2.6 inhibited the transcriptional activation capacity of *FtbZIP85* to *FtDFR*.

When comparing the treated and untreated hairy roots of Tartary buckwheat using HPLC, we found that the contents of epicatechin gallate (ECG), epicatechin (EC), epigallocatechin gallate (EGCG) and procyanidin B2 were significantly reduced following ABA treatment (50 μM or 75 μM) (Figure 5B). To analyze the role of *FtbZIP85* in the PA biosynthesis pathway, a hairy root culture system was established (Supplemental Figure S4). Subsequently, we generated overexpressing *FtbZIP85* hairy root lines: OE#1/#2/#3. The RT-qPCR analysis revealed that the expressions of *FtbZIP85*, *FtDFR*, *FtSnRK2.6, FtANS* and *FtLAR* were increased in OE#1/#2/#3 (Figure 5C). Therefore, the overexpression of *FtbZIP85* promoted the expressions of the PA biosynthesis-related genes. To further confirm this finding, we treated the TB seedlings with 100 μM of ABA. The results show that the contents of the PA-related substances (EC, procyandin B2 and procyandin B3) significantly decreased in the presence of ABA (Figure 5D). Hence, we infer that ABA is involved in PA biosynthesis by regulating the transactivation of *FtDFR*.

## 4. Discussion

Currently, most studies on the involvement of Tartary buckwheat transcription factors in flavonoid synthesis mostly focus on R2R3 MYB TFs [5,34], and there are few studies on the bZIP TF family in Tartary buckwheat PA biosynthesis. In addition, there are few studies on the influence of the flavonoid synthesis pathway substances in Tartary buckwheat acting via interactions with the important response genes of the ABA pathway. Only two bZIP TFs have been preliminarily noted to interact with SnRK2s [22,32]. In this study, we reported a novel bZIP TF, FtbZIP85, that positively regulates PA biosynthesis.

### 4.1. FtbZIP85 Played a Positive Role in PA Biosynthesis

In terms of transcription factor regulation, the synthesis of PA is mainly regulated by the MYB-bHLH-WDR (MBW) complex. Out of the R2R3-MYB TFs identified in anthocyanin/PA synthesis, only *FaMYB1* in strawberry (*Fragaria* × *ananassa*), PhMYB27 in petunia (*hybrida Vilm.*) and *VvMYBC2-L3* and *VvMYBC2-L1* in grape (*Vitis vinifera* L.) act as co-inhibitors by competitively binding to bHLH proteins; *FtMYB8* in Tartary buckwheat can inhibit anthocyanin/proanthocyanidin accumulation [5,34]. The overexpression transgenic lines of *FtMYB18* decreased anthocyanin and PA accumulation by downregulating the expressions of *NtCHS* and *NtDFR* in *tobacco*; those of *AtDFR* and *AtTT12* in *Arabidopsis*; and those of *FtCHS*, *FtDFR* and *FtANS* in Tartary buckwheat hairy roots. A molecular interaction analysis showed that *FtMYB18* inhibited anthocyanin and PA synthesis by forming an MBW transcription complex with *FtTT8* and *FtTTG1* or an MYB-JAZ complex with FtJAZ1/-3/-4/-7 [35]. *FtMYB3* negatively regulates anthocyanin and PA biosynthesis, mainly by downregulating the expressions of *AtDFR*, *AtANS*, *AtBAN* and *AtTT13* in transgenic *A. thaliana*, which may depend on the interaction of *FtMYB3* with *FtbHLH/FtWD40* [7]. Overall, in the study of PA biosynthesis, it was found that MYB TFs play a vital role in the PA biosynthesis in plants [7], while the regulatory mechanisms of bZIP TFs in TB remain to be clarified. In this study, we isolated a new basic leucine zipper, basic leucine zipper 85 (*FtbZIP85*), from Tartary buckwheat, and demonstrated that *FtbZIP85* could bind to BZIP (ABRE) motifs in the promoter of *FtDFR* and activate its expression (Figure 2). Moreover, in the analysis of the differential expressions of *FtbZIP85* and flavonoid-biosynthesis-related genes in TB tissues, it was found that *FtbZIP85* had a higher expression than *FtF3H* and *FtFLS* (flavonol synthase) in fresh seeds (Figure 1). This indicates that, unlike *FtMYB3* and *FtMYB18*, which act as negative regulators in PA biosynthesis, *FtbZIP85* may play a different role in the regulation of PA biosynthesis [5,35].

### 4.2. FtbZIP85 Is Involved in the Regulation of PA Biosynthesis via Interaction with FtSnRK2.6

ABA was proved to regulate flavonoid biosynthesis by regulating transcription regulators [36,37], and SnRK2s play an important role in response to ABA [38,39,40]. In apple, MdSnRK1.1 (sucrose nonfermenting 1 related kinase) targets and degrades MdJAZ18 to free MdbHLH, which leads to the activation of anthocyanin and proanthocyanidin accumulation [41]. An analysis of the phylogenetic tree of FtSnRK2s indicated that FtSnRK2.6 and FtSnRK2.2/2.3 may play differential functions in TB, consistent with the functional differentiation of AtSnRK2.6 in *A. thaliana* [42]. Furthermore, we found an interaction between FtSnRK2.6 and FtZIP85, and we found that FtSnRK2.6 phosphorylated FtZIP85 in vitro (Figure 4), which is consistent with FtZIP5 being a substrate of FtSnRK2.6. 

With the development of the biological industry in recent years, the cost of metabolome, transcriptome and genome research has greatly reduced. Hairy root cultures are being gradually applied to the study of metabolic pathways [43,44,45,46,47]. Nonetheless, our results indicate that ABA inhibited PA biosynthesis. However, the overexpression of FtbZIP85 in hairy roots promoted the biosynthesis of PA. Thus, we inferred that ABA regulates PA biosynthesis possibly by inducing the expression of FtSnRK2.6, which would further phosphorylate FtbZIP85. The phosphorylation of FtZIP85 would inhibit the transactivation capacity of FtbZIP85, which, in turn, would reduce the expression of *FtDFR*. Subsequently, the suppression of FtDFR inhibited PA biosynthesis. Taken together, our results suggest that ABA modulates PA biosynthesis possibly by inducing the action of FtSnRK2.6 to further phosphorylate FtbZIP85, which inhibits the transactivation capacity of FtbZIP85 on *FtDFR*.

## 5. Conclusions

Tartary buckwheat (Fagopyrum tataricum) is strongly adapted to growth in adverse environments [48]. bHLH TFs have been shown to participate in the regulation of flavonoid biosynthesis, and the local expression of bHLH promotes the expression of DFR and that of downstream enzymes in the anthocyanin biosynthetic pathway [34,49]. The activity of the regulatory proteins can be regulated via post-translational modifications, such as phosphorylation and dephosphorylation, to affect flavonoid biosynthesis [50,51]. Our study showed that the expressions of *FtDFR*, *FtbZIP85* and *FtSnRK2.6* were tissue-specific and that they were located in the nucleus and the cytosol. FtbZIP85 could positively regulate PA biosynthesis by regulating the transactivation of *FtDFR*, and FtbZIP85 was involved in the regulation of PA biosynthesis via an interaction with FtSnRK2.6. We speculate that ABA could activate FtSnRK2.6 phosphatase to further phosphorylate FtbZIP85, thereby inhibiting the transactivation capacity of FtbZIP85 to *FtDFR*. However, we have no direct evidence that ABA-activated FtSnRK2.6 reduces the expression of FtbZIP85. This is a weakness of our study and one of the difficulties in functional omics studies of TB as a non-model plant. Further functional studies involving transgenesis and mutant analyses should be established to further study the molecular mechanisms involved in the ABA signaling pathways associated with the regulation of PA biosynthesis. Flavonols occur as glycosides with numerous postulated biological roles in plants, including photoprotection, the modulation of hormone translocation and the sequestration of reactive oxygen species [52]. Under ABA stress, TB increases the antioxidant capacity of flavonoids and the content of endolysates to improve drought resistance, and, in this study, we provide some evidence for the role of ABA in the PA synthesis pathway.

Taken together, our data indicate that FtbZIP85 is a bZIP activator that positively regulates PA accumulation by strongly activating the expression of *FtDFR*. Therefore, FtbZIP85 is an important transcription factor in PA biosynthesis. Furthermore, we found that FtbZIP85 can directly interact with FtSnRK2.6 and that it was phosphorylated in vitro. Our study provides new insights for further research on the synthesis mechanism of TB PAs, and it is conducive to further research on the molecular mechanism of the synthesis regulation of TB PAs.

## Data Availability

Not applicable.

## Supplementary Materials

The following supporting information can be downloaded at: https://www.mdpi.com/article/10.3390/cimb45040221/s1, Figure S1: Conserved motifs of the key genes’ protein in TB flavonoid biosynthesis pathway were identified using MEME software (Multiple Em for Motif Elicitation). Figure S2: Supplemental Figure of Y1H and EMSA experiments. Figure S3: Phylogenetic analysis. Figure S4: Transgenic detection of *FtbZIP85* in TB hairy roots. Figure S5: Schematic diagram of *FtbZIP85* function in the regulation of anthocyanin and PA synthesis under ABA treatment. Table S1: Genes primers used in this study. Table S2: Key genes in flavonoid synthesis.
