# Supplementary material for: FtbZIP85 Is Involved in the Accumulation of Proanthocyanidin by Regulating the Transcription of FtDFR in Tartary Buckwheat
Source: Curr Issues Mol Biol. 2023 Apr 13;45(4):3375–90. doi: 10.3390/cimb45040221 (PMC10136674; doi:10.3390/cimb45040221)
Supplement: Supplementary file 1 [file cimb-45-00221-s001.zip › cimb-2227968-supplementary.pdf]

Supplemental Figures

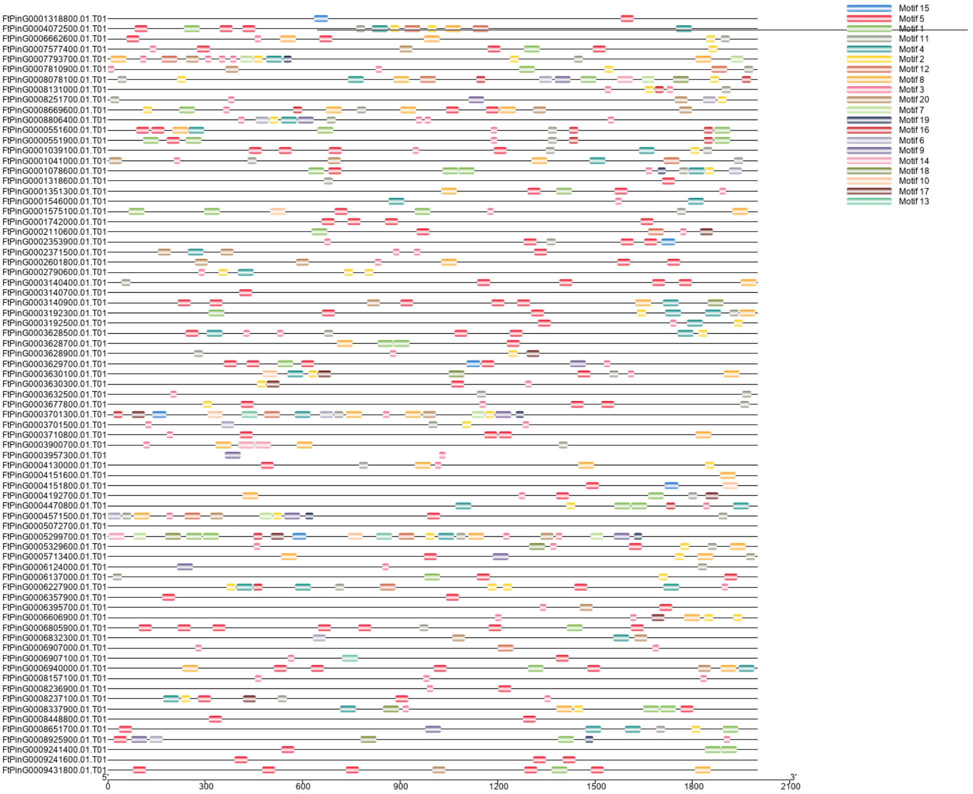

Figure S1. Conserved motifs of the key genes' protein in TB flavonoid biosynthesis pathway were identified using MEME software (Multiple Em for Motif Elicitation). Grey lines represent non-conserved sequences, and the length of lines proportionally represent the number of amino acids for each protein. Each motif is indicated with a color box numbered at the right. The length of the motifs in each protein is shown proportionally.

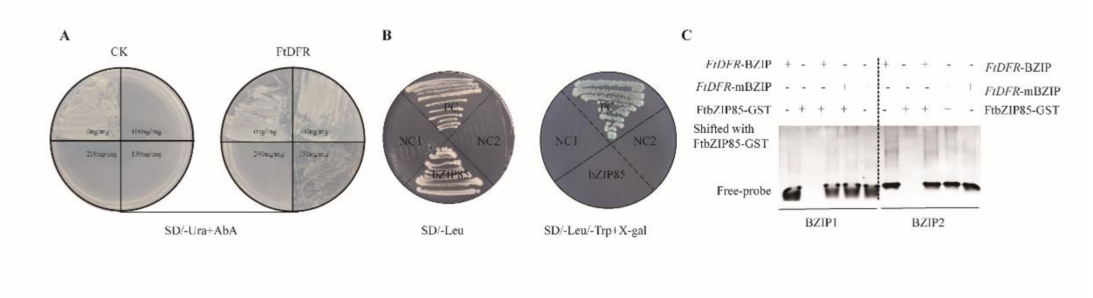

Figure S2. Supplemental Figure of Y1H and EMSA experiments. **A**, 200 ng/mL AbA can inhibit the growth of the FtDFR-pAbAi. **B**, FtbZIP85 has no autonomous activation activity. **C**. EMSA showing the mBZIP1and mBZIP2 has no specific binding of FtbZIP85 to the BZIP in the *FtDFR* promoter. The normal and mutant probes (m) were labeled with biotin. Unlabeled intact probes were used for competition. FtDFR-BZIP, competing probes not labeled with biotin. FtDFR-mBZIP, mutated competing probes not labeled with biotin. The red arrows indicate the shifted band representing the protein–DNA complex. The plus (+) and minus (-) signs denote the presence or absence of the probe or protein.

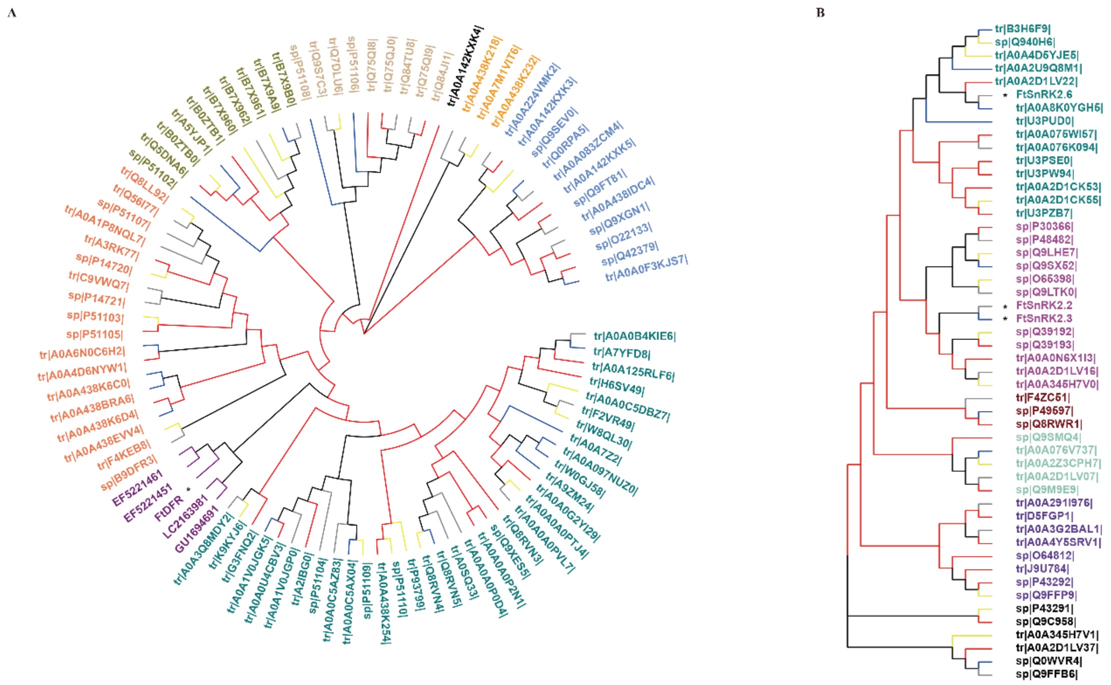

Figure S3. Phylogenetic analysis. A, Phylogenetic analysis of *FtDFRs* and other *DFRs* from different plant species. B. Phylogenetic analysis of *FtSnRK2.2/2.3/2.6* and other *SnRKs* from different plant species. Different branches genes were marked with different colors. * Marked with *FtDFR*, *FtSnRK2.2/2.3/2.6* presence or absence of the probe or protein.

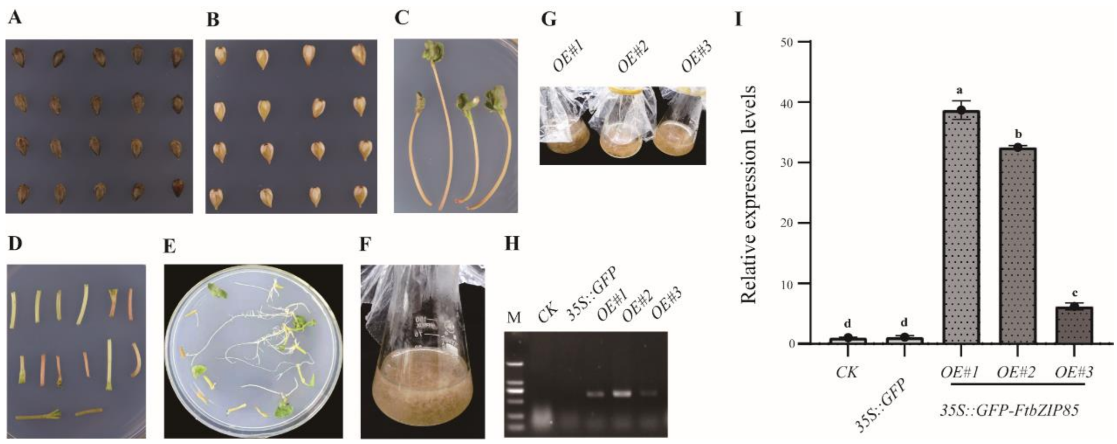

Figure S4 Transgenic detection of *FtbZIP85* in TB hairy roots. Representative images of critical stages are displayed: (A) and (B) represent before and after peeling off the seed coats; (C) denotes the seedlings of TB at 7~10 days after inoculation, and the part we used; (D) symbolize coculturing with activated *A. rhizogenes* and selective culturing on medium; (E) hairy roots emerge from; and (F) and (G) shows the propagation of hairy root formation; (H) and (I) shows the identification of hairy roots by PCR and RT-qPCR.

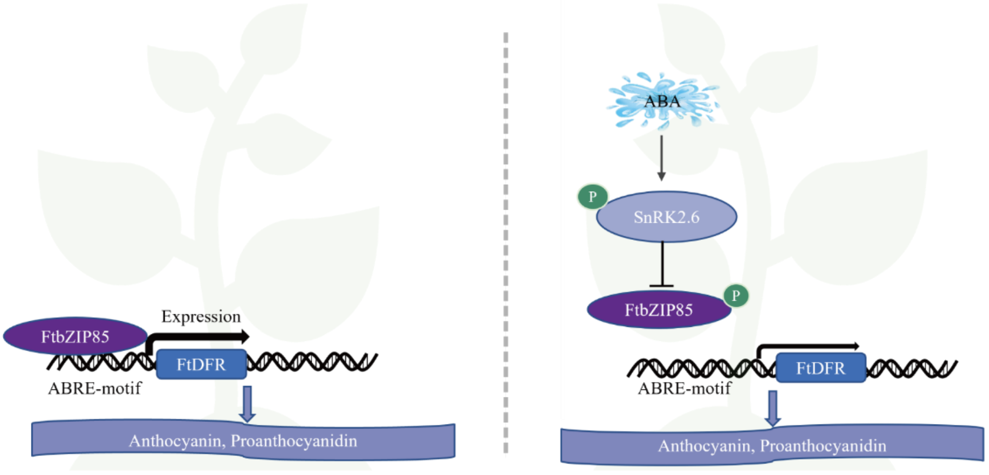

Figure S5. Schematic diagram of FtbZIP85 function in the regulation of anthocyanin and PA synthesis under ABA treatment. FtbZIP85 could positively regulate PA biosynthesis through regulating the transactivation of *FtDFR*. In ABA treatment, FtbZIP85 directly interacts with FtSnRK2.6 and is phosphorylated, which may inhibit the transcriptional activation of FtbZIP85 to *FtDFR*.

Supplementary Tables

Table S1. Genes primers used in this study.

Primers for Subcellular localization assays	pBI121-eGFP-FtbZIP85F	gcgtcgacATGATGAGGAACAGAGAGTCTGC
	pBI121-eGFP-FtbZIP85R	ggggtaccAAATAAGGCTGACAAGGATCGC
	pGT-mCherry-FtDFRF	gctctaga ATGGTTGCTGAGGGAGAGATC
	pGT-mCherry-FtDFRR	tcccccgggATGGCCATTACCAT
	pGT-mCherry-FtSnRK2.6F	gctctagaATGGATCGGCCGGCTGGG
	pGT-mCherry-FtSnRK2.6R	tccccccgggCATTGCATAGACTATCTCCCCGCT
	pGT-mCherryF	CCGCTCGAGATGGTGAGCAAGGGCGAGGAGG
	pGT-mCherryR	TGCTCTAGATTACTTGTACAGCTCGTCCATG
Primers for Y1H assays	BbZIP-F	CCTCGAGGTTGGAGTTTGTTGAGAACTTGAGT
	BbZIP-R	GCGGTACCGGTGCGACGTCGTTTTGG
	pGADT7-FtbZIP85F	CCGAATTCATGATGAGGAACAGAGAGTCTGC
	pGADT7-FtbZIP85R	CGGGATCCCCCCCCTCGAGGGTACC

	pGBKT7-FtbZIP85F	CCGAATTCATGATGAGGAACAGAGAGTCTGC
	pGBKT7-FtbZIP85R	CCGTCGACAAATAAGGCTGACAAGGATCGC
	pABAI-F	GTTCCTTATATGTAGCTTTCGACA
	pABAI-R	CCATCTCGAAAAAGGGTTTGCC
	pAbAr-F	GAAGATCCAAGGTGCGGG
	pURA3-R	TACGACCGAGATTCCCGG
Primers for EMSA assays	pGST-FtbZIP85F	CCGAATTCATGATGAGGAACAGAGAGTCTGC
	pGST-FtbZIP85R	CCGTCGACAAATAAGGCTGACAAGGATCGC
	BbZIP1-F	ATGAAACGGCCACCAGAATCTTACGTGGTCTACCGTCCGCTTCAC
	BbZIP1-R	GTGAAGCGGACGGTAGACCACGTAAGATTCTGGTGGCCGTTTCAT
	BbZIP1-MUT-F	ATGAAATTGCTACTAGAATCTTATTTGTTCTACTTTCTTCTTCAC
	BbZIP1-MUT-R	GTGAAGAAGAAAGTAGAACAAATAAGATTCTAGTAGCAATTTCAT
	BbZIP2-F	TATATATCTAACAAGTAAATCTAAGTACAAACACCTAA
	BbZIP2-R	TTAGGTGTTTGTACTTAGATTTACTTGTTAGATATATA
	BbZIP2-MUT-F	TATATATTTAACAATTAAATATAAGTATAAACACTTAA
	BbZIP2-MUT-R	TTAAGTGTTTATACTTATATTTAATTGTTAAATATATA
	BbZIP3-F	CAGCACACGTGCTACCAGTCCATAATCACGTGCC

		TAAATCTTCCACAGC
	BbZIP3-R	GCTGTGGAAGATTTAGGCACGTGATTATGGACTGGTAGCACGTGTGCTG
	BbZIP3-MUT-F	CATTACATTTGTTACTAGTCTATAATCACTTGTCTAAATCTTTCACATC
	BbZIP3-MUT-R	GATGTGAAAGATTTAGACAAGTGATTATAGACTAGTAACAAATGTAATG
	BbZIP4-F	CCGTATCTACAACAACAACGTGTATATATTAATTCTACCGTCCACACCAAA
	BbZIP4-R	TTTGGTGTGGACGGTAGAATTAATATATACACGTTGTTGTTGTAGATACGG
	BbZIP4-MUT-F	CTTTATCTACAATAATAACTTGTATATATTAATTCTACTTTCTACATCAAA
	BbZIP4-MUT-R	TTTGATGTAGAAAGTAGAATTAATATATACAAGTTATTATTGTAGATAAAG
Primers for LUC assays	pGreenII 0800-LUC-promoter-FtDFR-F	gcgtcgacGTTGGAGTTTGTTGAGAACTTGAGT
	pGreenII 0800-LUC-promoter-FtDFR-R	cgggatccGGTGCGACGTCGTTTTGG
	pCAMBIA1300-eGFP-FtbZIP85F	gcgtcgacATGATGAGGAACAGAGAGTCTGC
	pCAMBIA1300-eGFP-FtbZIP85R	cgggatcc CCCCCCTCGAGGGTACC
	pCAMBIA1307-4myc-FtSnRK2.6F	cgggatccATGGATCGGCCGGCTGGG
	pCAMBIA1	gcgtcgacCATTGCATAGACTATCTCCCCGCT

	307-4myc-FtSnRK2.6R	
	pGreenII 0800-LUC-F	GCGTCGACAAATGTTGTTCCTGCTAGAGAAATTGT
	pGreenII 0800-LUC-R	CATGCCATGGGCTCCTCCGCAAATTCTGAC
Primers for Y2H assays	pGADT7-FtbZIP85F	ccgaattc ATGATGAGGAACAGAGAGTCTGC
	pGADT7-FtbZIP85R	cgggatcc CCCCCCTCGAGGGTACC
	pGBKT7-FtSnRK2.6F	catgccatggATGGATCGGCCGGCTGGG
	pGBKT7-FtSnRK2.6R	gcgtcgacCATTGCATAGACTATCTCCCCGCT
	pGBKT7-FtSnRK2.3F	catgccatggATGGATCCGATGATGAATCG
	pGBKT7-FtSnRK2.3R	tccccccgggTAATGCATAGACAATCTCGCC
	pGBKT7-FtSnRK2.2F	catgccatggATGGATCCGATGATGAATCGGA
	pGBKT7-FtSnRK2.2R	gcgtcgacTATGGCATACACAATCTCCCC
Primers for BiFC assays	pSPYCE-FtSnRK2.6F	gctctagag ATGGATCGGCCGGCTGGG
	pSPYCE-FtSnRK2.6R	gcgtcgacCATTGCATAGACTATCTCCCCGCT
	pSPYNE-FtbZIP85F	gcgtcgacATGATGAGGAACAGAGAGTCTGC
	pSPYNE-FtbZIP8R	ggggtaccAAATAAGGCTGACAAGGATCGC
Primers for	pMal-p2x-MBP-	cggaatccATGGATCGGCCGGCTGGG

GST-pull down assays	FtSnRK2.6F	
	pMal-p2x-MBP-FtSnRK2.6R	gcgtcgacCATTGCATAGACTATCTCCCCGCT
	pGEX-6P-1-GST-FtbZIP85F	ccgaattcATGATGAGGAACAGAGAGTCTGC
	pGEX-6P-1-GST-FtbZIP85R	ccgtcgacAAATAAGGCTGACAAGGATCGC
Primers for Co-IP assays	pCAMBIA2300-5flag-FtSnRK2.6F	gctctagaATGGATCGGCCGGCTGGG
	pCAMBIA2300-5flag-FtSnRK2.6R	tccccccgggCATTGCATAGACTATCTCCCCGCT
	pBI121-eGFP-FtbZIP85F	gcgtcgacATGATGAGGAACAGAGAGTCTGC
	pBI121-eGFP-FtbZIP85R	ggggtaccAAATAAGGCTGACAAGGATCGC
Primers for qRT-PCR analysis	qFtbZIP85-F	AGAGTCTGCTGCTCGCTCTC
	qFtbZIP85-R	CCCTTCTCAGCCTGTCATTC
	qFtSnRK2.6-F	TGAGGATGAGGCTCGTTTCT
	qFtSnRK2.6-R	AAGCACCGACGACTTTGAGT
	qFtDFR-F	GGAGTGACGTTGACTTCTGC
	qFtDFR-R	GACTTGGAGGGAAACTTGGC
	qFtANR-F	ATGACCGGCCGTTCTATTAC

	qFtANR-R	CCAGACAGCATTTGCATACC
	qFtLAR-F	CGATGTACTTGGAGAAGCGA
	qFtLAR-R	GGTCCATAGGAGGTGTGACC
	qFtF3'H-F	GCTGGCGTATGCTTAGGAAG
	qFtF3'H-R	GGCCTTGGATAATGCCCTTG
	qFtF3H-F	AACAGCAGCCGTTTGTCAAT
	qFtF3H-R	TGCTCCTTAGCCAGCTTCTT
	qFtCHI-F	GCTCACGATCAATGGTGCAT
	qFtCHI-R	CGAATTGACCGGTGACAACA
	qFtCHS-F	TCTCGACAACCGTCAAGACA
	qFtCHS-R	GCACATGATGACGTGTGTGA
	qFt4CL-F	GCAACCATAGACGCTCAAGG
	qFt4CL-R	TGCATCGGCTATGGATGGAT
	qFtC4H-F	GTCCTGGTATCATCCTCGCA
	qFtC4H-R	GCGGTGGGCTTCATAACAAT
	qFtPAL-F	ACAAGGCGTTACATGGAGGA
	qFtPAL-R	CCAAGCTAGGGTTTCTCCCA
	qFtActin7-F	ATGTTCACTACCACCGCTGA
	qFtActin7-R	TGAACCTCTCAGCACCAATC

Table S2. Key genes in flavonoid synthesis.

Number	ID	Number	ID
1	FtPinG0001318800.01.T01	45	FtPinG0003957300.01.T01
2	FtPinG0004072500.01.T01	46	FtPinG0004130000.01.T01
3	FtPinG0006662600.01.T01	47	FtPinG0004151600.01.T01
4	FtPinG0007577400.01.T01	48	FtPinG0004151800.01.T01
5	FtPinG0007793700.01.T01	49	FtPinG0004192700.01.T01
6	FtPinG0007810900.01.T01	50	FtPinG0004470800.01.T01
7	FtPinG0008078100.01.T01	51	FtPinG0004571500.01.T01
8	FtPinG0008131000.01.T01	52	FtPinG0005072700.01.T01
9	FtPinG0008251700.01.T01	53	FtPinG0005299700.01.T01
10	FtPinG0008669600.01.T01	54	FtPinG0005329600.01.T01
11	FtPinG0008806400.01.T01	55	FtPinG0005713400.01.T01
12	FtPinG0000551600.01.T01	56	FtPinG0006124000.01.T01
13	FtPinG0000551900.01.T01	57	FtPinG0006137000.01.T01
14	FtPinG0001039100.01.T01	58	FtPinG0006227900.01.T01
15	FtPinG0001041000.01.T01	59	FtPinG0006270000.01.T01
16	FtPinG0001078600.01.T01	60	FtPinG0006357900.01.T01
17	FtPinG0001318600.01.T01	61	FtPinG0006395700.01.T01

18	FtPinG0001351300.01.T01	62	FtPinG0006606900.01.T01
19	FtPinG0001546000.01.T01	63	FtPinG0006662600.01.T01
20	FtPinG0001575100.01.T01	64	FtPinG0006805900.01.T01
21	FtPinG0001742000.01.T01	65	FtPinG0006832300.01.T01
22	FtPinG0002110600.01.T01	66	FtPinG0006832300.01.T01
23	FtPinG0002353900.01.T01	67	FtPinG0006907000.01.T01
24	FtPinG0002371500.01.T01	68	FtPinG0006907100.01.T01
25	FtPinG0002601800.01.T01	69	FtPinG0006907100.01.T01
26	FtPinG0002790600.01.T01	70	FtPinG0006940000.01.T01
27	FtPinG0003140400.01.T01	71	FtPinG0008131000.01.T01
28	FtPinG0003140700.01.T01	72	FtPinG0008157100.01.T01
29	FtPinG0003140900.01.T01	73	FtPinG0008236900.01.T01
30	FtPinG0003192300.01.T01	74	FtPinG0008237100.01.T01
31	FtPinG0003192500.01.T01	75	FtPinG0008251700.01.T01
32	FtPinG0003628500.01.T01	76	FtPinG0008337900.01.T01
33	FtPinG0003628700.01.T01	77	FtPinG0008448800.01.T01
34	FtPinG0003628900.01.T01	78	FtPinG0008651700.01.T01
35	FtPinG0003629700.01.T01	79	FtPinG0008806400.01.T01
36	FtPinG0003630100.01.T01	80	FtPinG0008925900.01.T01
37	FtPinG0003630300.01.T01	81	FtPinG0008925900.01.T01

38	FtPinG0003632500.01.T01	82	FtPinG0009022800.01.T01
39	FtPinG0003677800.01.T01	83	FtPinG0009071200.01.T 01
40	FtPinG0003701300.01.T01	84	FtPinG0009241400.01.T01
41	FtPinG0003701500.01.T01	85	FtPinG0009241600.01.T01
42	FtPinG0003710800.01. T01	86	FtPinG0009431800.01.T01
43	FtPinG0003710800.01.T01	87	FtPinG0009894100.01.T01
44	FtPinG0003900700.01.T01		
